# Comparative effect of vortioxetine and sertraline on clinical and inflammatory profile in Parkinson’s disease with comorbid depression

**DOI:** 10.3389/fnins.2026.1761550

**Published:** 2026-01-22

**Authors:** Marika Alborghetti, Camilla Moliterni, Edoardo Bianchini, Domiziana Rinaldi, Daniela Caissutti, Antonella Capozzi, Fabiana Giada Radicati, Antonella Moschillo, Martina Marino, Isabella Berardelli, Marco Salvetti, Ferdinando Nicoletti, Roberta Misasi, Francesco Ernesto Pontieri

**Affiliations:** 1 Department of Neurosciences, Mental Health, and Sensory Organs, Sapienza University of Rome, Rome, Italy; 2 IRCCS Neuromed, Pozzilli, Italy; 3 Center for Parkinson’s Disease, IRCCS San Raffaele Pisana, Rome, Italy; 4 Department of Experimental Medicine, Sapienza University of Rome, Rome, Italy; 5 AGEIS, Université Grenoble Alpes, Grenoble, France; 6 Department of Physiology and Pharmacology, Sapienza University of Rome, Rome, Italy

**Keywords:** cognitive impairment, inflammation, major depressive disorder, Parkinson’s disease, vortioxetine

## Abstract

**Background:**

Preclinical and clinical studies suggest that the multimodal antidepressant vortioxetine may offer a valuable therapeutic option in the treatment of depression associated with neurological disorders, including Parkinson’s disease (PD).

**Objectives:**

To compare the effect of vortioxetine and the SSRI sertraline in the treatment of PD and comorbid depression, placing emphasis on peripheral inflammation markers.

**Methods:**

This is an observational longitudinal study. We have recruited 33 patients affected by PD with comorbid depression, evaluated for motor and non-motor symptoms, depression, and peripheral and soluble markers of inflammation at baseline and at the end of antidepressant treatment. Patients were divided into two groups treated with either vortioxetine (10–20 mg/day) or sertraline (50 mg/day) for 4 months. A group of 12 healthy controls was also used for comparative purposes.

**Results:**

At baseline PD patients showed a higher number of “classical” monocytes and a lower number of “non-classical” monocytes and myeloid dendritic cells (mDCs). The number and activity of DCs generated from isolated monocytes were also lower in PD patients. While both sertraline and vortioxetine treatment further reduced the number of mDCs, only vortioxetine had a restorative effect on CD40- and CD54-expressing DCs and their function. Both drugs display an antidepressant activity, but vortioxetine was superior to sertraline in improving cognitive function, anxiety, anhedonia, and apathy.

**Conclusion:**

These data evaluate for the first time the immunomodulatory effects of two different treatments in PD patients with comorbid depression, highlighting the greater immunomodulatory capacity of vortioxetine.

## 1 Introduction

Depression occurs in PD more frequently than in the general population, with an average prevalence of 35–38% (Reijnders et al., 2008; Cong et al., 2022). A recent meta-analyses on 129 studies has shown that depression in PD is more prevalent in women, in patients carrying the glucocerebrosidase mutation, and is frequently associated with freezing of gait (FOG), apathy, anxiety, and fatigue. The prevalence of depression increases with the progression of PD and has a negative impact on motor and non-motor signs and quality of life (Cong et al., 2022).

Drug treatment of depression in PD is challenging. In some patients depressive symptoms are markedly improved in response to conventional dopaminergic medications (e.g., L-DOPA and/or dopamine receptor agonists). However, in most cases, this treatment is suboptimal, and the addition of an antidepressant is required. An optimal antidepressant medication in PD should combine a good efficacy in improving depressive mood, anhedonia, apathy, anxiety, cognitive dysfunction and sleep disorders, a good profile of safety and tolerability, and a lack of interference with typical motor signs of PD. For example, the use of tricyclic antidepressants, which display a high efficacy in improving depressive mood, is limited by the off-target effects, which may cause cognitive impairment and orthostatic hypotension (Ulrich et al., 2020). The SSRI sertraline is one of the most commonly used antidepressants in PD, owing to its ability to inhibit dopamine reuptake at high doses and to its favourable PK profile (Hauser and Zesiewicz, 1997). However, similarly to other SSRIs, sertraline may cause sexual dysfunction, gastrointestinal adverse effects, increases in body weight, and emotional blunting.

The multimodal antidepressant, vortioxetine, is emerging as a potential candidate drug in the treatment of depression in PD (Sanchez et al., 2015; Sowa-Kućma et al., 2017). Vortioxetine is a multitarget agent, inhibiting the high-affinity serotonin transporter with a target saturation >80%, behaving as a full agonist of 5-HT1_A_ receptors, a partial agonist of 5-HT1_B_ receptors, a potent antagonist of 5-HT3 receptors, and a weaker antagonist of 5-HT1_D_ and 5-HT7 receptors (Sanchez et al., 2015). Vortioxetine shows superiority with respect to all other antidepressants in the treatment of cognitive dysfunction associated with MDD (Baune et al., 2018; Lam et al., 2024; Vigod et al., 2025), displays a good therapeutic effect against anhedonia and emotional blunting (Fagiolini et al., 2021), shows a good profile of safety and tolerability (Vigod et al., 2025), and is not an inhibitor or inducer of any isoform of cytochrome P450. *In vitro* data have shown that vortioxetine was able to drive differentiation of human monocytes into M2-type macrophages, characterized by an anti-inflammatory phenotype (Talmon et al., 2018). Furthermore, vortioxetine has been reported to inhibit type 1 and 2 cyclooxygenase (COX-1/2) with a surprisingly high potency (Talmon et al., 2020). SSRIs have also consistently shown anti-inflammatory action in preclinical studies, although data with sertraline are not uniform (Mariani et al., 2022).

Neuroinflammation is involved in the pathophysiology of both PD and major depressive disorder (MDD), as shown by histological analyses of post-mortem brain tissues, PET-imaging with tracers targeting brain-activated microglia, and evaluation of immune cells and cytokines/chemokines in the peripheral blood and cerebralspinal fluid (Tansey et al., 2022; Drevets et al., 2022; Nashtahosseini et al., 2025). MDD has been associated with increased levels of peripheral biomarkers of inflammation, a blunted adaptive immune response, and microglia activation in the CNS (Drevets et al., 2022; Moshfeghinia et al., 2025). A causal link between inflammation and MDD is suggested by the evidence that individuals with high levels of pro-inflammatory markers are more prone to develop depression later in life (Bell et al., 2017; Lamers et al., 2019). Interestingly, biological drugs targeting immune cells are under clinical development for the treatment of MDD (Raison et al., 2013; Tiosano et al., 2020). The role of neuroinflammation in PD is well established and is reflected by changes in immune-competent cells in the peripheral blood (Williams-Gray et al., 2016; Lindqvist et al., 2012). There is evidence that the amount of circulating monocytes is altered in patients affected by PD, and their hyperactivity correlates with disease severity. “Classical” CD14^+^/CD16^−^ inflammatory monocytes are increased in PD patients, while “non-classical” CD14^+^/CD16^+^ monocytes, involved in regenerative processes and integrity of the vessel wall, are reduced (Grozdanov et al., 2014). The increased “classical” monocytes in patients affected by PD have been related to the risk of developing cognitive impairment (Wijeyekoon et al., 2020). Dendritic cell (DC) levels were shown to be reduced in PD, with reduction being inversely related to motor impairment (Ciaramella et al., 2013). Finally, higher levels of blood pro-inflammatory cytokines have been reported in PD (Wijeyekoon et al., 2020; Ciaramella et al., 2013).

To our knowledge, there are no studies examining the impact of antidepressant treatment on peripheral markers of inflammation in patients affected by PD. In this observational study, we compared the efficacy of a 4-month treatment with either sertraline or vortioxetine on depressive symptoms, anxiety, apathy, cognitive function, and the immunological and inflammatory phenotype in patients with PD and comorbid depression.

## 2 Methods

### 2.1 Participants and drugs

This study received approval from the local Ethics Committee (Protocol No. 49 SA_2022, RIF. CE 6652_2021), and was conducted in accordance with the Human Rights Principles adopted by the World Medical Association at the 18th WMA General Assembly, Helsinki, Finland, June 1964, and subsequently amended by the 64th WMA General Assembly, Fortaleza, Ceará, Brazil, October 2013. Informed consent was obtained from all participants.

The study was carried out on subjects diagnosed with idiopathic PD, according to the MDS criteria (Postuma et al., 2015), recruited consecutively during routine visits at the Outpatient Service for Movement Disorders of the Sant’Andrea University Hospital, Department NESMOS, Sapienza University of Rome. Upon the report of depressive symptoms, subjects were submitted to a psychiatric visit to confirm comorbidity with MDD, according to the DSM-5 criteria (American Psychiatric Association, 2013). All subjects were under stable antiparkinsonian therapy and did not show changes in the severity of motor symptoms for at least 4 months before enrollment. Exclusion criteria were as follows: cognitive impairment as measured by a Mini Mental State Examination score <24; other psychiatric or neurological disorders; active treatment with anti-inflammatory drugs; active treatment with non-selective monoamine-oxidase (MAO) or MAO_A_ inhibitors; treatment with immunosuppressant/immunomodulatory drugs in the last 12 months; active infectious or inflammatory diseases; presence or history of-neoplasms; hematologic disorders; chronic obstructive pulmonary disease or asthma; hepatic or renal failure; coronary syndrome, cardiomyopathy, heart failure; unstable hypertension; diabetes; autoimmune diseases; history of traumatic brain injury in the last 12 months; history of drug or alcohol abuse.

At baseline visit, subjects were submitted to the following rating scales:

- the MDS Unified Parkinson’s Disease Rating Scale-part III (MDS-UPDRS-III) (Postuma et al., 2015) for quantification of the severity of motor symptoms of parkinsonism;
- the Beck Depression Inventory (BDI) (Levin et al., 1988) as a self-reported questionnaire to assess the severity of depressive symptoms;
- the Hamilton Depression Rating Scale (HAM-D) (Weintraub et al., 2006) as a clinician-administered tool for the quantitative assessment of the severity of depressive symptoms and the track treatment response;
- the Hamilton Anxiety Rating Scale (HAM-A) (Leentjens et al., 2011) for identification and measurement of symptoms of anxiety;
- the Snaith-Hamilton Pleasure Scale (SHAPS) (Martino et al., 2018) for the identification of anhedonia;
- the Apathy Evaluation Scale (AES) (Leentjens et al., 2008) for the identification and measurement of apathy;
- the Stroop Word Color Test (SWCT) (Scarpina and Tagini, 2017) for the assessment of cognitive interference, the difficulty in processing information when there is a conflict between two stimuli;
- the Trail Making Test A (TMT-A) and B (TMT-B) (Arbuthnott and Frank, 2000) for the assessment of visual attention, task switching, and executive functions;
- the Digit Symbol Substitution Test (DSST) (Jaeger and Zaragoza, 2016) for the evaluation of short term, verbal memory and working memory.

At the end of the evaluation scales, subjects were submitted to a venous blood sample for the laboratory tests indicated below. They were then prescribed either sertraline or vortioxetine based on the decision of the senior neurologist and psychiatrist, taking into account factors such as patient comorbidities, previous treatment history, potential drug interactions, and tolerability profiles. Sertraline was prescribed at a daily dose of 50 mg, according to the summary of product characteristics (SmPC). Vortioxetine was prescribed as oral drops and titrated over a 10/20-day period to achieve the dose of 10, 15, or 20 mg, according to treatment response.

Rating scales and laboratory testing were repeated after 16 weeks of treatment with sertraline or vortioxetine (final visit). Adverse events, treatment compliance, and the patient’s status were further assessed through follow-up phone calls 1 and 2 months after the treatment initiation.

### 2.2 Neuroinflammatory state

#### 2.2.1 Isolation of peripheral blood mononuclear and dendritic cells

Peripheral blood samples were obtained by venepunctures, collected into 10-mL EDTA tubes (EDTAVacutainer, BD Biosciences, San Diego, CA, United States), processed, and stained within 4 h of collection. An aliquot of each sample was centrifuged at 2,000 × g for 10 min at room temperature (18–25 °C). Plasma was carefully aspirated, collected, and stored at −80 °C until further analysis. Freshly collected whole blood was diluted with sterile phosphate-buffered saline (PBS, Aurogene Srl, Italy) at a 1:3 ratio (1 part blood to 3 parts PBS), and peripheral blood mononuclear cells (PBMC) were isolated by centrifugation on a density gradient following the manufacturer’s instructions (Lymphoprep, Gentaur Srl, Italy). After centrifugation, PBMCs were collected and washed twice in washing buffer (PBS containing 0.5% FBS and 2 mM EDTA) by centrifugation at 300 × g for 10 min at room temperature. PBMCs were stained and analyzed by flow cytometry. Monocyte subpopulations were evaluated by the expression of specific surface markers: CD14^+^/CD16^−^ monocytes (“classical”) and CD14^+^/CD16^+^ monocytes (“non-classical”).

The percentage of peripheral blood DCs was assessed by measuring membrane marker expression of CD11c (myeloid DCs or mDCs) or CD123 (plasmacytoid DCs or pDCs).

#### 2.2.2 Generation of monocyte-derived DCs

Monocyte-derived DCs (moDCs) were obtained by PBMC using anti-CD14 monoclonal antibodies conjugated to magnetic beads, followed by MACS column separation (Miltenyi Biotec, Germany) (Grozdanov et al., 2014). Monocytes were suspended in RPMI 1640 (Sigma-Aldrich, United States) complete medium supplemented with 50 ng/mL GM-CSF and 10 ng/mL IL-4 (Miltenyi Biotech, Germany), seeded at 1 × 10^6^ cells/mL in 6-well plates (Sarsted, Germany), and incubated for 8 days at 37 °C with 5% CO_2_. These cells constitute the immature DCs. On day 6, to induce maturation of moDCs, half of the wells were stimulated with 200 ng/mL of LPS (Merck Life Science, Germany) for the last 48 h of culture. On day 8, both immature and mature cells were analyzed, and supernatants were stored until use. Cell counts and viability were evaluated using trypan blue (Sigma-Aldrich, United States) exclusion method and confirmed by flow cytometry.

#### 2.2.3 Evaluation of moDCs maturation

To evaluate the maturation of moDCs, the expression of CD40 and CD54 (ICAM-1), as well as endocytic activity was assessed by flow cytometry. Endocytic activity was evaluated by measuring the uptake of fluorescein isothiocyanate-carboxymethyl-dextran (FITC-dextran, Merck Life Science, Germany). moDCs were incubated for 1 h with 1 mg/mL FITC-dextran at +37 °C. Negative control was obtained by incubating mature moDCs with FITC-dextran for 1 h at +37 °C. After incubation, cells were fixed with 2% formaldehyde in the dark for 20 min at +37 °C. Cells were then washed with PBS, and FITC-dextran uptake was quantified as mean fluorescence intensity (MFI). Ten thousand cells of each sample were analyzed by flow cytometry (CytoFLEX, Beckman Coulter, United States).

#### 2.2.4 Flow cytometry staining strategy and cytofluorimetric analysis

The following antibody panels were used for flow cytometric analysis: FITC anti-CD16 (B49215, clone 3G8), PC5.5 anti-CD14 (A70204, clone RMO52), PC5.5 anti-CD11c (B19719, clone BU15), PC7 anti-CD123 (B13647, clone SSDCLY107D2), PE anti-CD40 (IM1936U, clone MAB89), FITC anti-CD54 (IMO726U, clone 84H10). To determine surface cell phenotype, cells were incubated with the appropriate antibodies for 40 min at +4 °C. After incubation, cells were washed and then analyzed by flow cytometry. All antibodies were obtained from Beckman Coulter (United States). Measurement of the expression of surface markers was performed by multiparametric analysis by flow cytometry (CytoFLEX, Beckman Coulter, United States). Data were analyzed by CytExpert software and expressed as MFI or percentage of positive cells.

#### 2.2.5 Cytokine assays

The patients’ inflammatory profile was assessed by measuring IFN-γ, IL-10, TNF-α, IL-1β, IL-6, and TGF-β cytokines in cell culture supernatants and plasma. Levels of IFN-γ, IL-10, TNF-α, and IL-1β were determined using the Luminex Discovery Assay (Human Premixed Multi-Analyte kit, R&D Systems, United States). Levels of IL-6 were measured by BD OptEIA^™^ ELISA Kit II (BD Biosciences, United States), and TGF-β was determined using the Quantikine ELISA kit (R&D Systems, United States).

### 2.3 Statistical analyses

All statistical analyses were performed using RStudio v2022.02.2 + 433 for Windows (R Foundation for Statistical Computing, Vienna, Austria). Descriptive statistics and frequency tables were calculated for the examined variables. Normality of distribution of variables was assessed using the Shapiro–Wilk test. In the case of a non-normal distribution, a rank transformation was performed. To assess the clinical difference between the two groups of treatment al BL and the laboratory features between PD patients and HD, Student’s *t*-test was used. To assess the difference between the treatment group at BL and after 4 months of treatment, two-way ANOVAs for repeated measures were used, with the factors “time” and “group.” *Post-hoc t*-tests with Bonferroni’s correction of *p*-values were performed in case of significant ANOVA main effects. If necessary, Greenhouse–Geisser’s correction for non-sphericity was applied. The significance threshold was set at *α* < 0.05. All data were reported as mean ± SD (Q1–Q3) for numerical data and *N* (%) for categorical variables. Only patients who completed both baseline and follow-up evaluations were included in the longitudinal analyses. No imputation for missing data was performed.

## 3 Results

Demographic data are reported in Table 1. We enrolled 33 patients with PD and comorbid depression (20 males and 13 females; age = 68 ± 8 years). Sixteen patients (11 male and 5 females) were treated with sertraline, and 17 patients (9 males and 8 females) with vortioxetine. Sertraline was administered orally at the dose of 25 mg q.d. in the first week of treatment, and then the indicated dose of 50 mg q.d. in all patients. Vortioxetine was titrated from 1 mg orally q.d. at day one, increasing the dose of 1 mg per day up to 10 (*n* = 9), 15 (*n* = 5), or 20 (*n* = 3) mg q.d. depending on the response of the patients.

**Table 1:** Clinical features of the PD population under treatment with sertraline or vortioxetine.

	Age	Sex	Age at onset	Disease duration	HeY	Phenotype	Motor fluctuation	LEED
PD population	68 ± 8 years	20 males 13 females	60 ± 8 years	7.5 ± 3 years	1/1.5: 3 patients 2/2.5: 23 patients 3: 7 patients	TD: 14 patients PIGD: 19 patients	No: 17 patients Yes: 16 patients	550 mg
								Sertraline	73 ± 6 years	11 males 5 females	65 ± 6.5 years	8 ± 3 years	1/1.5: — 2/2.5: 13 patients 3: 3 patients	TD: 7 patients PIGD: 9 patients	No: 10 patients Yes: 6 patients	600 mg
																Vortioxetine	64 ± 7.5 years	9 males 8 females	56 ± 8 years	7 ± 3.5 years	1/1.5: 3 patients 2/2.5: 10 patients 3: 4 patients	TD: 7 patients PIGD: 10 patients	No: 7 patients Yes: 10 patients	500 mg

PD, Parkinson’s disease; HeY, Hoehn and Yahr stage; TD, tremor-dominant phenotype; PIGD, postural instability and gait disturbance; LEDD, L-DOPA equivalent daily dose.

Age and age at onset of PD were slightly but significantly shorter (*p* = 0.04) in the group of patients treated with vortioxetine. The other clinical features did not differ between the two groups of patients.

Two patients discontinued treatment with sertraline due to the occurrence of moderate agitation and insomnia after one week of treatment. Three patients discontinued treatment with vortioxetine due to the occurrence of unmanageable nausea in the first week of treatment, and one patient due to concerns of adverse events during the first week of treatment.

### 3.1 Differential effect of sertraline and vortioxetine treatment on depression, cognitive profile, anxiety, anhedonia, and apathy

The severity of motor symptoms assessed by subscale III of MDS-UPDRS did not change after treatment with either vortioxetine (28.9 vs. 28.5 points) or sertraline (32.7 vs. 35 points).

**Figure 1:**
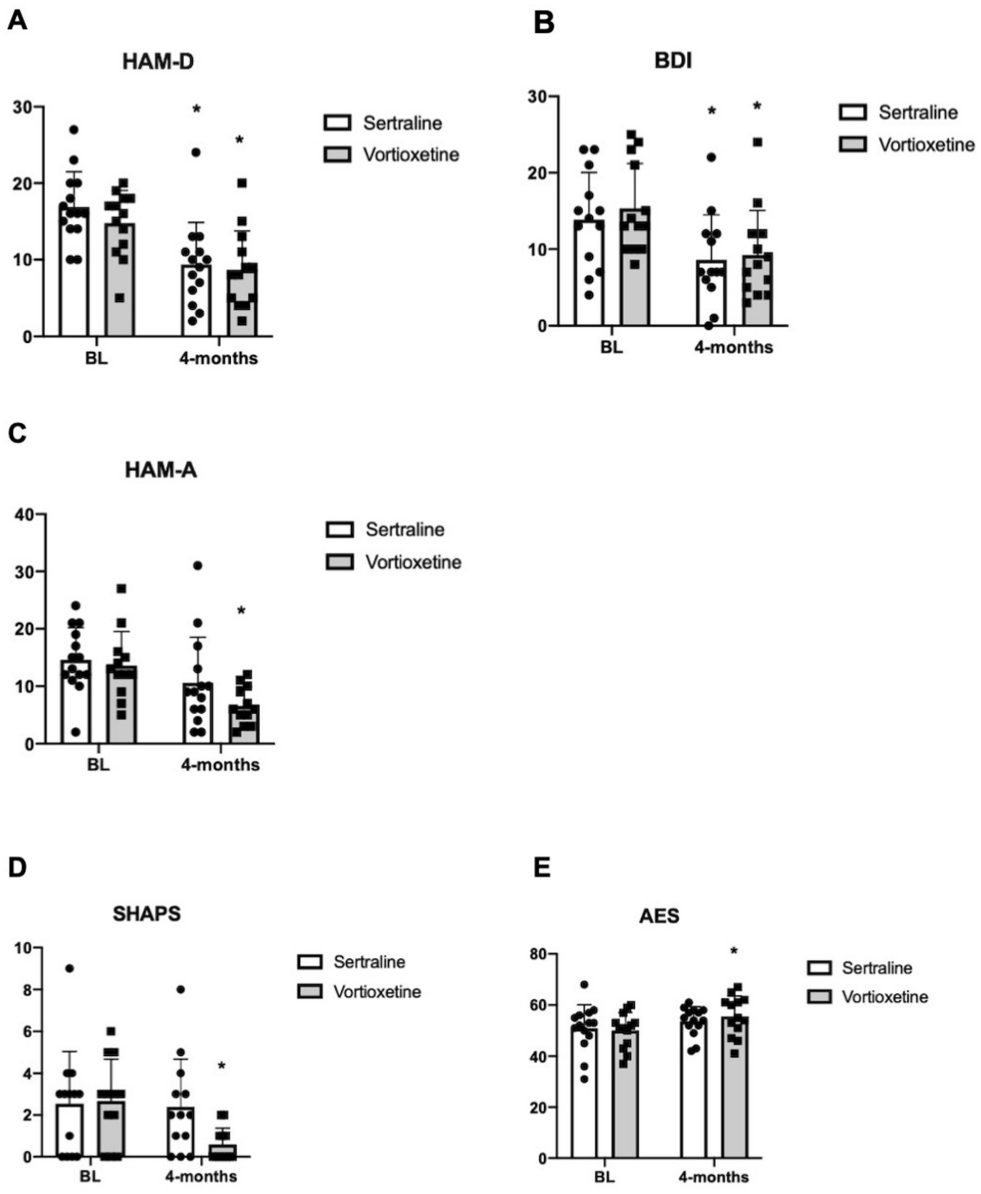
Depression, anxiety, and anhedonia evaluated in subgroups of patients under treatment with sertraline and vortioxetine. BDI and HAM-D (means ± SEM) for the evaluation of depression in PD patients at BL and after 4 months of treatment with sertraline and vortioxetine are shown in **A,B**, respectively. HAM-A (means ± SEM) for the evaluation of anxiety in PD patients at baseline (BL) and after 4 months of treatment with sertraline and vortioxetine are shown in **C**. SHAPS (means ± SEM) for the evaluation of anhedonia in PD patients at BL and after 4 months of treatment with sertraline and vortioxetine are shown in **D**. AES (means ± SEM) for the evaluation of apathy in PD patients at BL and after 4 months of treatment with sertraline and vortioxetine are shown in **E**. Two-way ANOVA for repeated measures + Bonferroni. ^*^p < 0.05 vs. the respective BL. Statistically significant outliers were identified using ROUT (alpha = 0.05) and excluded from further analysis.

As shown in Figure 1, the two drugs did not differ in improving depressive symptoms as assessed by HAM-D and BDI. With the HAM-D scale, 12/13 patients treated with vortioxetine and 14/14 treated with sertraline had a BL score ≥8. After 4 months of treatment, patients with persistence of depressive symptoms were 5/13 and 7/14 in the groups of vortioxetine and sertraline, respectively. The reduction in average HAM-D score was from 15 to 8 points (*p* = 0.002) in the vortioxetine group and from 16 to 9 points in the sertraline group (*p* = 0.0002) (Figure 1A). With the BDI scale, 13/13 patients treated with vortioxetine and 10/14 treated with sertraline had a BL score ≥7. After 4 months of treatment, patients with persistence of depressive symptoms were 6/13 and 6/14 in the groups of vortioxetine and sertraline, respectively. The reduction in average BDI score was from 15 to 9 points (*p* < 0.001) in the vortioxetine group and from 17 to 10 points in the sertraline group (*p* = 0.005) (Figure 1B).

Only vortioxetine treatment was able to improve anxiety as assessed by the HAM-A scale. At BL, 12/13 and 13/14 patients showed anxiety (HAM-A ≥8) in the groups of vortioxetine and sertraline, respectively. After 4 months of treatment with vortioxetine or sertraline, patients with persistence of anxiety symptoms were 5/13 and 9/14, respectively. Treatment with vortioxetine significantly reduced the HAM-A score (*p* = 0.004) (Figure 1C).

Assessment of apathy and anhedonia also showed a remarkable difference between vortioxetine and sertraline. At BL, 8/13 and 9/14 patients showed anhedonia (SHAPS ≥ 3) in the vortioxetine and sertraline group, respectively. After 4 months of treatment, patients with persistence of anhedonia were 1/13 and 5/14 in the vortioxetine and sertraline group, respectively. Only treatment with vortioxetine significantly improved the SHAPS score after 4 months (*p* = 0.01) (Figure 1D). Similar findings were obtained using the AES for measurement of apathy (Figure 1E). AES score displayed a significant, although moderate, improvement only in the vortioxetine group (from 50 to 56 points after 4 months of treatment; *p* = 0.005). Sertraline treatment induced no changes in the AES score (from 51 to 53 points).

**Figure 2:**
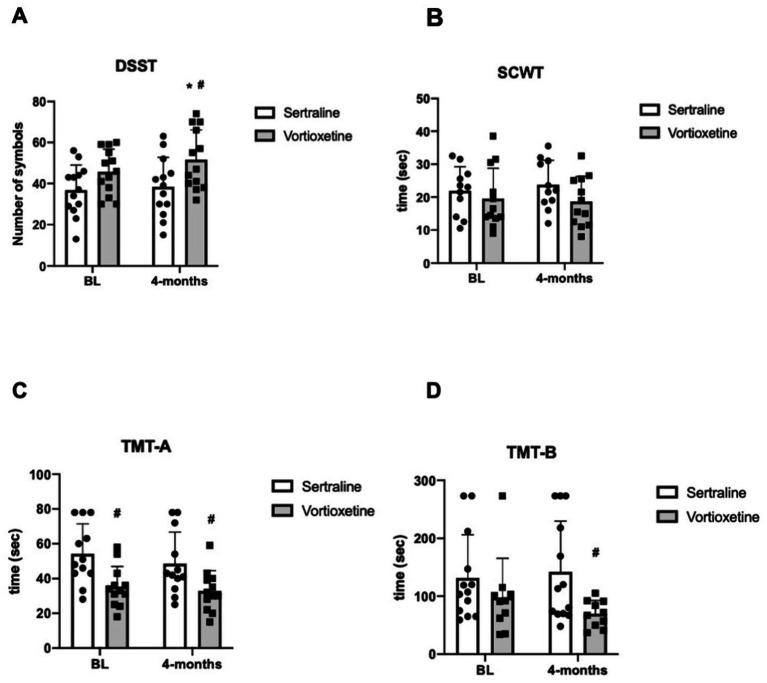
Cognitive performance in subgroups of patients under treatment with sertraline and vortioxetine. DSST, SCWT, TMT-A, and TMT-B (means ± SEM) for the evaluation of cognition in PD patients at baseline (BL) and after 4 months of treatment with sertraline and vortioxetine are shown in **A–D**, respectively. Mixed ANOVA, ^*^p < 0.05 vs. sertraline, ^#^*p* < 0.05 vs. the respective BL. Two-way ANOVA for repeated measures + Bonferroni. ^*^p < 0.05 vs. the respective BL, ^#^*p* < 0.05 vs. sertraline. Statistically significant outliers were identified using ROUT (alpha = 0.05) and excluded from further analysis.

Vortioxetine improved cognitive function evaluated with the DSST and TMT-B scales, but not with the SCWT and TMT-A scales (Figures 2A–D). In the DSST, treatment with vortioxetine resulted in a higher number of inserted symbols, increasing from 45 at BL to 52 after 4-months of treatment (Figure 2A). Vortioxetine treatment significantly improved patients’ performance in the TMT-B test (from 104.4 s at BL to 73.1 s after 4-months of treatment; *p* = 0.03) (Figure 2D). Sertraline failed to improve cognitive function. The difference in DSST between vortioxetine and sertraline at 4 months of treatment was markedly significant (52 vs. 38 symbols, *p* = 0.02) (Figure 2A).

### 3.2 PD patients showed a differential monocyte and DC phenotype expression compared to controls

In order to make a comparison on the inflammatory profile, 12 blood samples from healthy donor (HD) subjects, matched for age and sex with patients, were included in the study (7 males and 5 females; age = 61 ± 5.3 years).

Since classical monocyte enrichment in blood has been observed in PD patients (Grozdanov et al., 2014), we first investigated different monocyte subpopulations in peripheral blood from PD patients and age-matched HD subjects. As expected, classical monocytes were significantly higher in PD patients (*p* = 0.03), while non-classical monocytes were significantly lower (0.03) (Supplementary Figures 1A,B).

mDCs and pDCs were identified by CD11c or CD123 markers, respectively. Both subpopulations were lower in PD patients, with only the difference in mDCs being statistically significant (*p* = 0.02) (Supplementary Figures 1C,D). However, patients with PD associated with postural instability and gait disorder (PIGDs) showed an increased percentage of pDCs compared to patients with a tremor-dominant phenotype (0.94% vs. 0.52%; *p* = 0.01).

**Figure 3:**
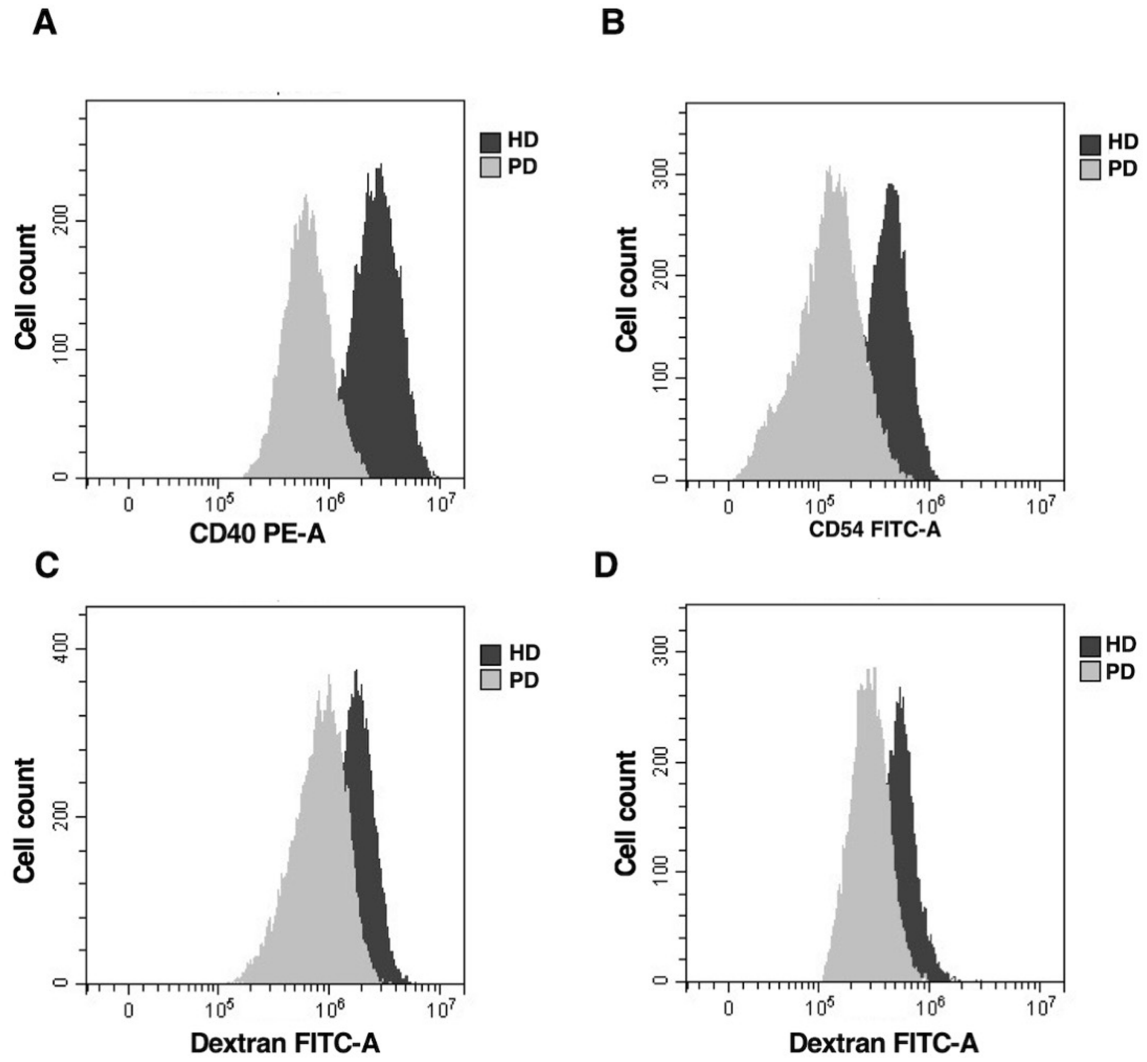
Comparable expression of phenotype markers and antigen uptake capability in moDCs derived from PD patients and HD subjects. Flow cytometric analysis of CD40 **(A)** and CD54 **(B)** molecules expression on mature moDCs from one representative PD patient or HD subject. FITC dextran endocytosis on immature **(C)** and mature **(D)** moDCs from one representative PD patient or HD subject.

The co-stimulatory molecule CD40 was upregulated in mature moDCs in both PD patients and HD (Figure 3A and Table 2). However, the intensity differed between the two groups, with CD40 expression being significantly lower in PD (*p* = 0.01). Moreover, both in PD and HD, mature moDCs expressed higher levels of CD54 molecule than immature moDCs, but the levels were significantly lower in PD patients (*p* = 0.03) (Figure 3B and Table 2). As expected, the endocytosis efficiency of immature moDCs, in PD and HD, was significantly higher than that of mature moDCs (*p* < 0.0001). Furthermore, mature moDCs from PD patients almost completely lose their ability to uptake antigens (*p* < 0.0001) (Figures 3C,D and Table 2).

**Table 2:** Expression of surface molecules in immature and mature moDCs from PD and HD.

Cells	Group	CD40	CD54	Dextran FITC
Immature moDCs	HD (*n* = 12)	138.6 ± 83.1	13.7 ± 10.2	171.5 ± 34.8
	PD (*n* = 28)	73.5 ± 38.2^**^	12.0 ± 5.4	40.3 ± 19.9^****^
Mature moDCs	HD (*n* = 12)	246.3 ± 225.7	56.3 ± 35.2	41.2 ± 5.6
	PD (*n* = 28)	101.0 ± 38.5^**^	37.9 ± 14.6^*^	11.6 ± 7.3^****^

moDCs, monocyte-derived dendritic cells. Expression of each molecule is presented as mean fluorescence intensity. Data are expressed as mean values ± SD; the statistical significance is shown as compared to HD, ^*^p < 0.05, ^**^p < 0.01, ^***^p < 0.001, and ^****^p < 0.0001.

These data suggest that *in vitro* cultured moDCs are capable of differentiation and maturation, although PD-derived moDCs may exhibit impaired costimulatory capacity and antigen-presenting cell (APC) function.

To assess pro-inflammatory features of moDCs, the levels of various pro- and anti-inflammatory cytokines were measured in plasma and cell culture supernatants. The concentration of IL-1β, IL-6, and TNF-α, released by mature moDCs from PD patients, was significantly higher than that from the HD (Supplementary Figures 2A,B). Interestingly, the molecules IFN-γ and IL-10 were significantly reduced in mature moDC supernatants of PD patients (Supplementary Figure 2C), while no statistically significant differences between the two groups were observed regarding TGF-β release (Supplementary Figure 2D). In contrast, plasma levels of these cytokines showed much less pronounced differences between the two groups, which is consistent with findings reported in literature (Supplementary Figures 3A–D). This result may reflect a low-grade inflammatory environment in PD patients.

### 3.3 Differential effect of sertraline and vortioxetine treatment on the inflammatory profile in patients affected by PD and comorbid depression

**Figure 4:**
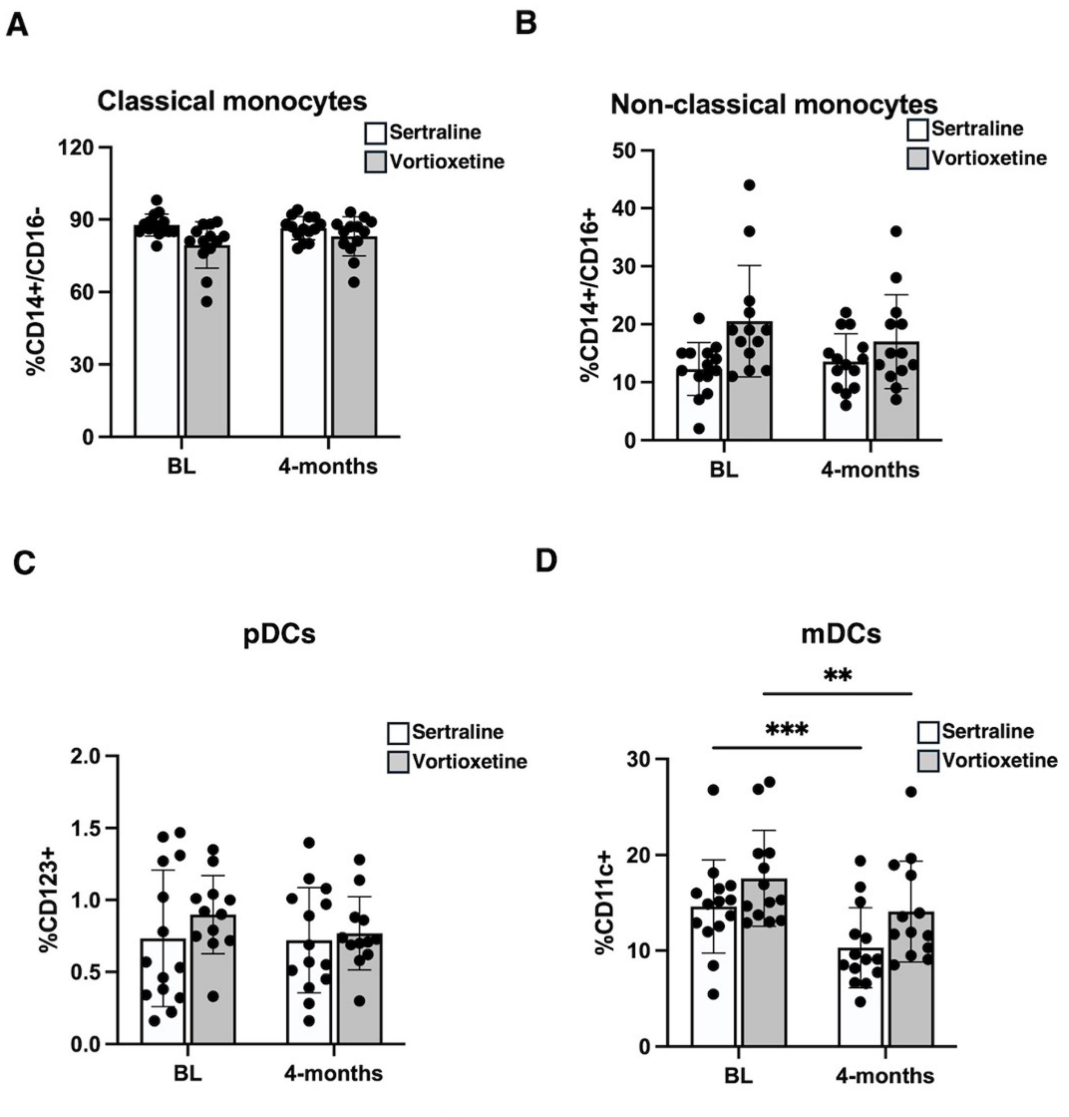
Differential effect of sertraline and vortioxetine on peripheral blood mononuclear cells (PBMCs) of PD patients. PBMCs were isolated from blood samples of PD patients treated with sertraline or vortioxetine and were analyzed, at baseline (BL) and after 4 months of treatment, to investigate monocyte and DC subpopulations. Cells were evaluated, by cytofluorimetric analysis, for the expression of specific surface markers, identifying: CD14^+^/CD16^−^ as classical monocytes **(A)**, CD14^+^/CD16^+^ as non-classical monocytes **(B)**, CD123^+^ as plasmacytoid DCs (pDCs), and CD11c^+^ as myeloid DCs (mDCs). Data are reported as mean ± SD, ^**^p < 0.01 and ^***^p < 0.001.

Our results showed no significant differences in classical and non-classical monocytes after sertraline and vortioxetine treatments (Figures 4A,B). Additionally, while vortioxetine treatment led to a reduction in pDCs, sertraline treatment did not have this effect. However, both treatments caused a significant decrease in mDCs (*p* = 0.007 for vortioxetine, and *p* = 0.008 for sertraline) (Figures 4C,D).

**Figure 5:**
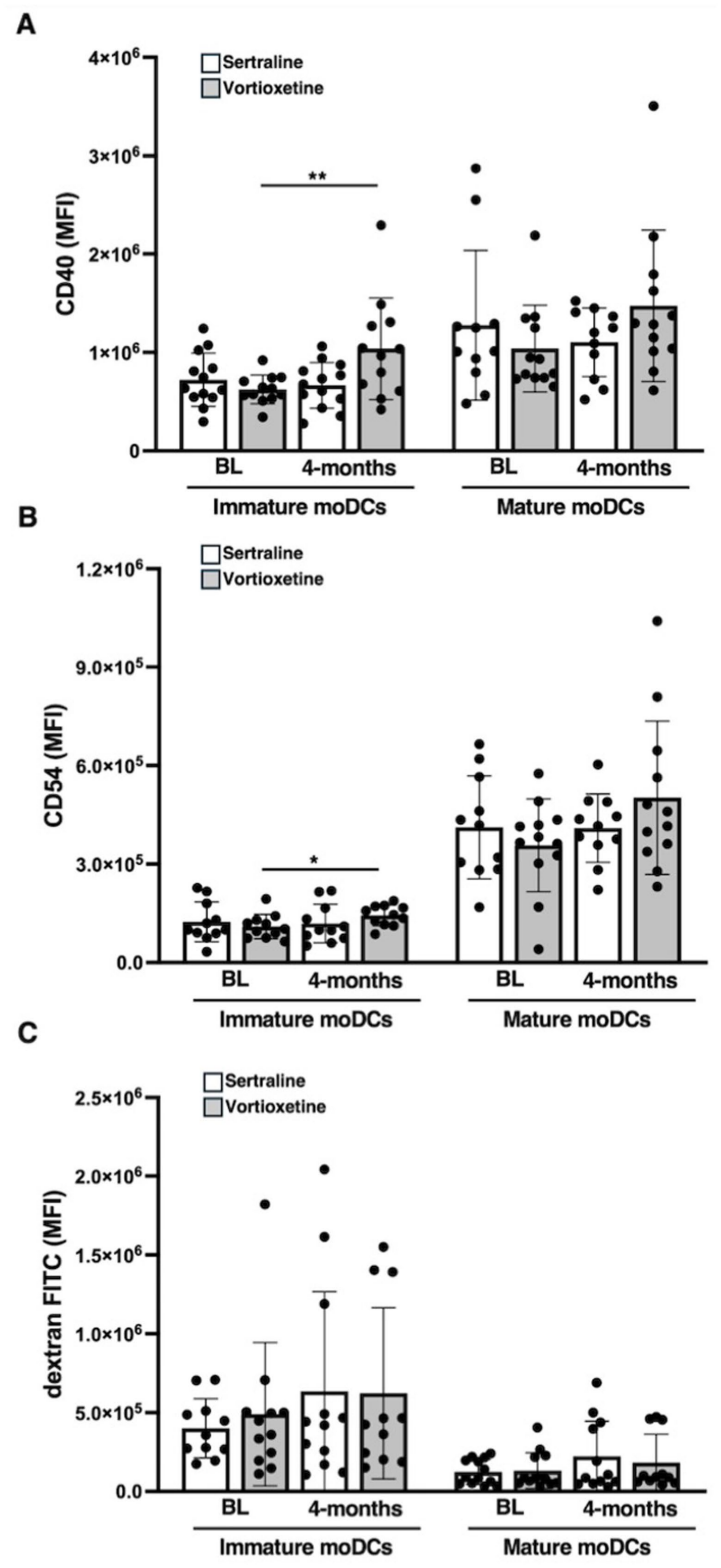
Comparison of sertraline or vortioxetine effect on moDCs functional properties in PD patients. Immature and mature moDCs from PD patients, at baseline (BL) and after 4 months of treatment with sertraline or vortioxetine, were analyzed by cytofluorimetric analysis to assess: CD40 **(A)** or CD54 **(B)** expression and moDCs antigen uptake capability by measuring FITC-dextran cell internalization **(C)**. Data are reported as mean ± SD, ^*^p < 0.05 and ^**^p < 0.01.

Vortioxetine treatment significantly increased CD40 expression in immature moDCs, compared to its BL (*p* = 0.005) and to sertraline treatment (*p* = 0.01) (Figure 5A). A trend toward increased CD40 expression was also observed in mature moDCs following vortioxetine treatment, while sertraline treatment resulted in an opposite trend (Figure 5A).

In immature DCs, vortioxetine, but not sertraline, significantly increased CD54 expression compared to BL values (*p* = 0.01) (Figure 5B). In mature moDCs, CD54 expression levels also showed a trend to increase following vortioxetine treatment (Figure 5B).

Although both sertraline and vortioxetine treatments reduced the ability of mature moDCs to internalize dextran FITC (Figure 5C), there was no difference compared with BL (Figure 5C).

These results suggest that vortioxetine may modulate the phenotype of PD-derived DCs, enhancing their costimulatory and APC capabilities.

**Figure 6:**
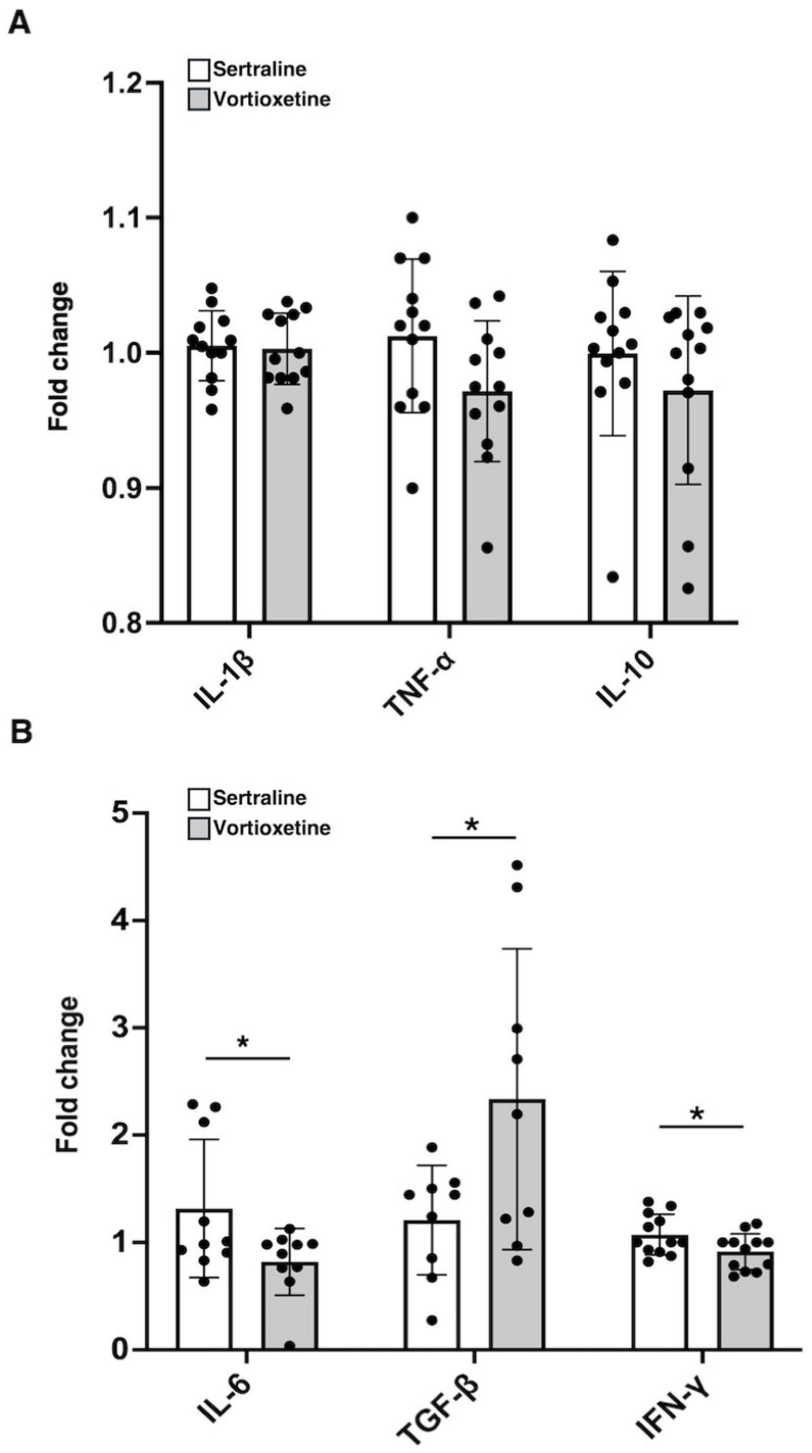
Plasma of PD patients was analyzed for cytokine levels. Evaluation of IL-1-β, TNF-α, IL-10 **(A)**, and IL-6, TGF-β, IFN-γ **(B)** levels in plasma of PD patients treated either with sertraline (*n* = 12) or vortioxetine (*n* = 12) was quantified by ELISA. Results are expressed as fold change with respect to their baseline. Data are reported as mean ± SD, ^*^p < 0.05.

Neither vortioxetine nor sertraline treatments affected plasma levels of IL-1β, TNF-α, or IL-10 (Figure 6A). On the contrary, IL-6, TGF-β, and INF-γ seem to be modulated by vortioxetine treatment (Figure 6B).

**Figure 7:**
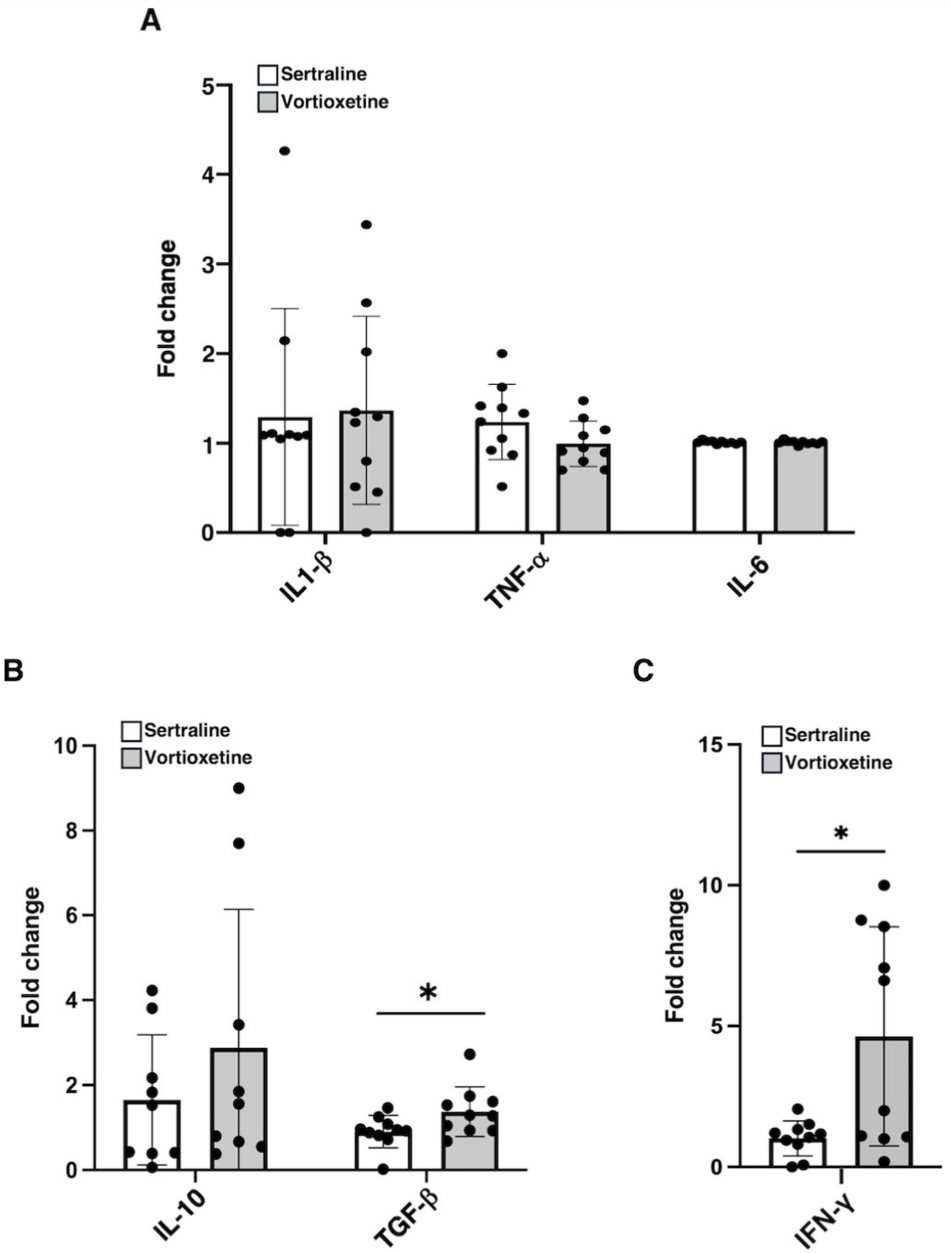
Supernatants from mature moDCs were analyzed for cytokine levels. Detection of IL-1-β, TNF-α, IL-6 **(A)**, IL-10, TGF-β **(B)**, and IFN-γ **(C)** levels in supernatants of mature DCs derived from monocytes of PD patients treated either with sertraline (*n* = 12) or vortioxetine (*n* = 12). Cytokines were quantified by ELISA. Results are expressed as fold change with respect to their baseline. Data are reported as mean ± SD, ^*^p < 0.05.

Levels of pro-inflammatory cytokines (IL-1β, TNF-α, and IL-6) secreted into the supernatant of mature moDCs did not show significant differences (Figure 7A). Notably, cytokines involved in inflammation resolution clearly showed significant changes, in particular, IL-10 and TGF-β as anti-inflammatory cytokines, and INF-γ as an important mediator for DC complete maturation (Figures 7B,C).

## 4 Discussion

To our knowledge, there are only two open-label prospective studies (Santos García et al., 2022; Stocchi et al., 2025) and a case report (Yoshimura et al., 2021) evaluating the efficacy of vortioxetine in the treatment of depression in patients affected by PD. In one study, a 12-week treatment with vortioxetine significantly improved depressive mood, fatigue, apathy, and cognition (Santos García et al., 2022). In the other study, a 16-week treatment with vortioxetine was well tolerated and significantly improved depressive symptoms without affecting motor symptoms of PD (Stocchi et al., 2025). In the case report described by Yoshimura et al. (2021), one patient with PD and comorbid depression, who did not respond to either paroxetine or escitalopram, was switched to the full dose of vortioxetine (20 mg/die) with a significant improvement of depressive symptoms (Yoshimura et al., 2021).

Here, we confirmed the efficacy of vortioxetine (at doses ranging from 10 to 20 mg/day) in improving depressive symptoms in patients affected by PD. This study has some limitations that should be acknowledged. First, the sample size was relatively small, which may reduce the statistical power and limit the generalizability of our findings. Second, treatment assignment was not randomized but based on clinical judgment. While this reflects real-world clinical practice, it may have introduced selection bias and confounding by indication, particularly since differences in non-motor symptom profiles could have influenced antidepressant choice. Consequently, causal inferences cannot be drawn from our results. Future randomized controlled trials including larger cohorts are warranted to more definitively confirm the differential clinical and immunomodulatory effects observed between vortioxetine and sertraline. However, the study has the merit of a head-to-head comparison between vortioxetine and sertraline, which is one of the most widely used antidepressants in PD (Hauser and Zesiewicz, 1997). We wish to highlight that the age at onset of PD was approximately nine years lower in the group of patients treated with vortioxetine, although disease duration was similar in the two groups. Despite this limitation, vortioxetine showed better efficacy in improving cognitive function, anxiety, and anhedonia, while the two drugs were equally efficacious in improving depressive symptoms evaluated with HAM-D and BDI scales. The greater effect of vortioxetine on cognition is in line with several reports in which vortioxetine was compared to antidepressants (Baune et al., 2018; Lam et al., 2024; Vigod et al., 2025). As opposed to sertraline, vortioxetine treatment induced a significant improvement in anxiety with respect to baseline values, and the HAM-A score was lower in vortioxetine-treated than in sertraline-treated patients, although the difference was not statistically significant. These findings are surprising, knowing the efficacy of SSRIs on anxiety disorders. Of note, 8 out of 17 vortioxetine-treated patients received doses of either 15 or 20 mg/day, which were found to be effective on anxiety evaluated with HAM-A (Baldwin et al., 2016; Adair et al., 2023). Full therapeutic doses of vortioxetine may relieve anxiety by recruiting 5-HT_1A_ receptors, which are known to be targeted by anxiolytic drugs (e.g., buspirone, ipsipirone, and gepirone) (Yocca, 1990; Kaur Gill et al., 2019). Only vortioxetine was able to significantly improve apathy in our patients, although there was no great difference in the AES score between sertraline and vortioxetine. In a recent report, SSRIs were found to worsen apathy in patients with PD (Schade et al., 2025). In patients with MDD, treatment with SSRIs and SNRIs may cause apathy/emotional blunting, and both manifestations are improved after switching to vortioxetine (Fagiolini et al., 2021; Fagiolini, 2024). Thus, vortioxetine might be a drug of choice in patients with PD and a high level of apathy.

We examined a series of peripheral markers of inflammation and innate immunity, as an indirect indication of neuroinflammation associated with both PD and depression (see Introduction and references therein). Consistent with previous findings (Wijeyekoon et al., 2020; Ciaramella et al., 2013), we found a significant increase in “classical” monocytes and a reduction in “non-classical” monocytes in the blood of patients affected by PD and comorbid depression, as compared to healthy controls. Levels of mDCs were significantly reduced in our patients, and levels of pDCs also showed a trend toward a reduction. In addition, DCs generated *in vitro* from monocytes isolated from patients with PD and depression showed a reduced number and antigen incorporation. mDCs and pDCs differ in their pattern of cytokine secretion and ability to drive T cell differentiation, and play a key role in both innate and adaptive immunity (Eisenbarth, 2019). Thus, our findings suggest an altered immunological profile in patients with PD and comorbid depression.

An important difference between vortioxetine and sertraline was that only vortioxetine was able to increase the percentage of CD40^+^ and CD54^+^ DCs generated from monocytes, suggesting that vortioxetine may have a restorative function on defective APCs in PD with comorbid depression. The most relevant finding was the different impact of sertraline and vortioxetine treatment on immunological profile (Eslami et al., 2025b) and the blood cytokine profile. Remarkably, vortioxetine reduced the levels of two pro-inflammatory cytokines, i.e., IL-6 and IFN-γ, and enhanced the levels of the anti-inflammatory cytokine, TGF-β, whereas sertraline caused no changes in cytokine levels. Increased IL-6 levels in blood have been consistently linked to MDD (reviewed by Drevets et al., 2022). For example, high blood IL-6 levels at age 9 years increased the risk of developing depression later in life (Khandaker et al., 2014), and high IL-6 levels predicted a chronic course of disease in women with major depression (Lamers et al., 2019). In addition, men with depression had higher IL-6 levels compared to healthy controls (Vogelzangs et al., 2012). In a meta-analysis evaluating >2,600 study participants, blood IL-6 levels were found to be significantly increased in patients affected by PD in most of the studies, whereas IFN-γ levels were unchanged (Qin et al., 2016), suggesting a potential role for IL-6 in the pathophysiology of PD. There are only a few studies on blood TGF-β levels in PD. TGF-β drives naïve T cell differentiation into T-regulatory (Treg) cells underlying immune tolerance (Moreau et al., 2022), and Treg cells and other regulatory cells were found to be defective in PD (Álvarez-Luquín et al., 2019). Future studies should also consider the role of systemic and gut–brain immune interactions (Eslami et al., 2025a).

This suggests that vortioxetine treatment may support immune tolerance in PD. These findings strengthen *in vitro* data showing that vortioxetine displays a potent anti-inflammatory activity (see Introduction and References therein), and might therefore restrain inflammation associated with both PD and depression.

Taken together, our study suggests that vortioxetine may be a valuable drug in the treatment of PD and comorbid depression owing to its powerful therapeutic activity on depressive symptoms, anxiety, apathy/emotional blunting, cognitive dysfunction, and inflammation. The drug has an optimal profile of safety and tolerability, with the exception of nausea, which can be managed with a slow titration. In addition, vortioxetine is not an inhibitor or inducer of any isoform of cytochrome-P_450_, and can be combined with other medications with low risk of PK interactions (Chen et al., 2018). Therefore, vortioxetine may be considered as a new valuable drug in the therapeutic armamentarium of depression associated with PD.

## Data Availability

The original contributions presented in the study are included in the article/supplementary material, further inquiries can be directed to the corresponding author.
