# Supplementary material for: Comparative effect of vortioxetine and sertraline on clinical and inflammatory profile in Parkinson’s disease with comorbid depression
Source: Front Neurosci. 2026 Jan 22;20:1761550. doi: 10.3389/fnins.2026.1761550 (PMC12874709; doi:10.3389/fnins.2026.1761550)
Supplement: Supplementary file 1 [file Data_Sheet_1.pdf]

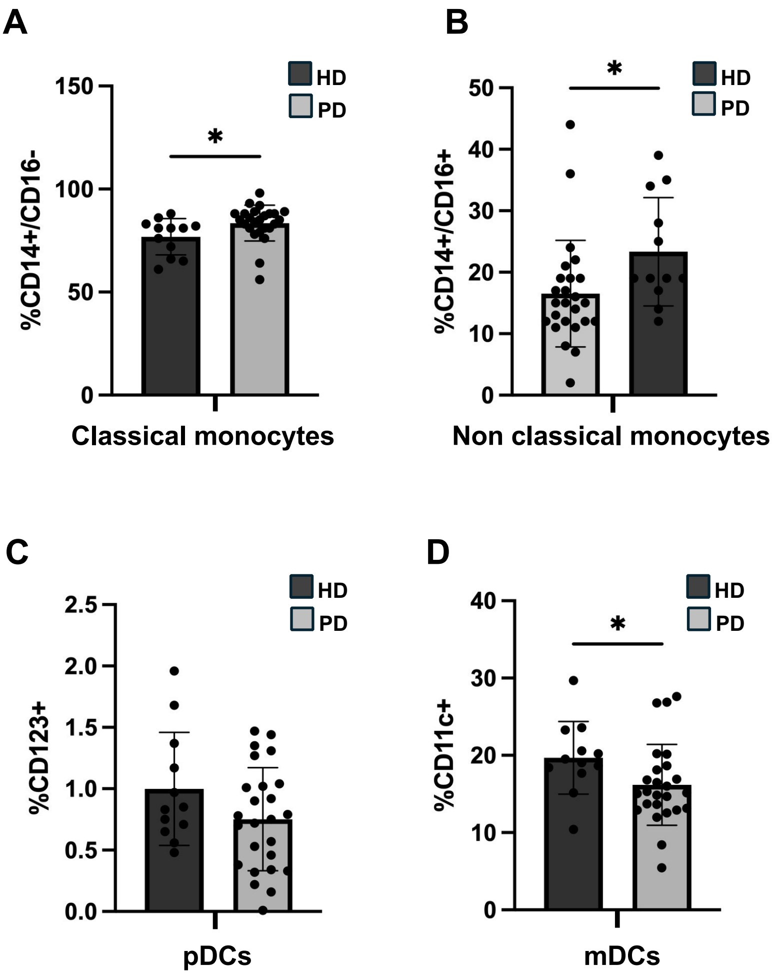

Figure S1 - Comparison of peripheral blood cell counts in PD patients and HD subjects.
PBMCs were isolated from blood samples of PD patients and HD subjects and then analyzed for monocyte and DC subpopulations. Cells were evaluated, by cytofluorimetric analysis, for the expression of specific surface markers, identifying: $CD14^{+}/CD16^{-}$ as classical monocytes (A), $CD14^{+}/CD16^{+}$ as non-classical monocytes (B), $CD123^{+}$ as plasmacytoid DCs (pDCs) and $CD11c^{+}$as myeloid DCs (mDCs). Data are reported as mean ± SD, * $p < 0.05$.
