# Supplementary material for: Comparative effect of vortioxetine and sertraline on clinical and inflammatory profile in Parkinson’s disease with comorbid depression
Source: Front Neurosci. 2026 Jan 22;20:1761550. doi: 10.3389/fnins.2026.1761550 (PMC12874709; doi:10.3389/fnins.2026.1761550)
Supplement: Supplementary file 2 [file Data_Sheet_2.pdf]

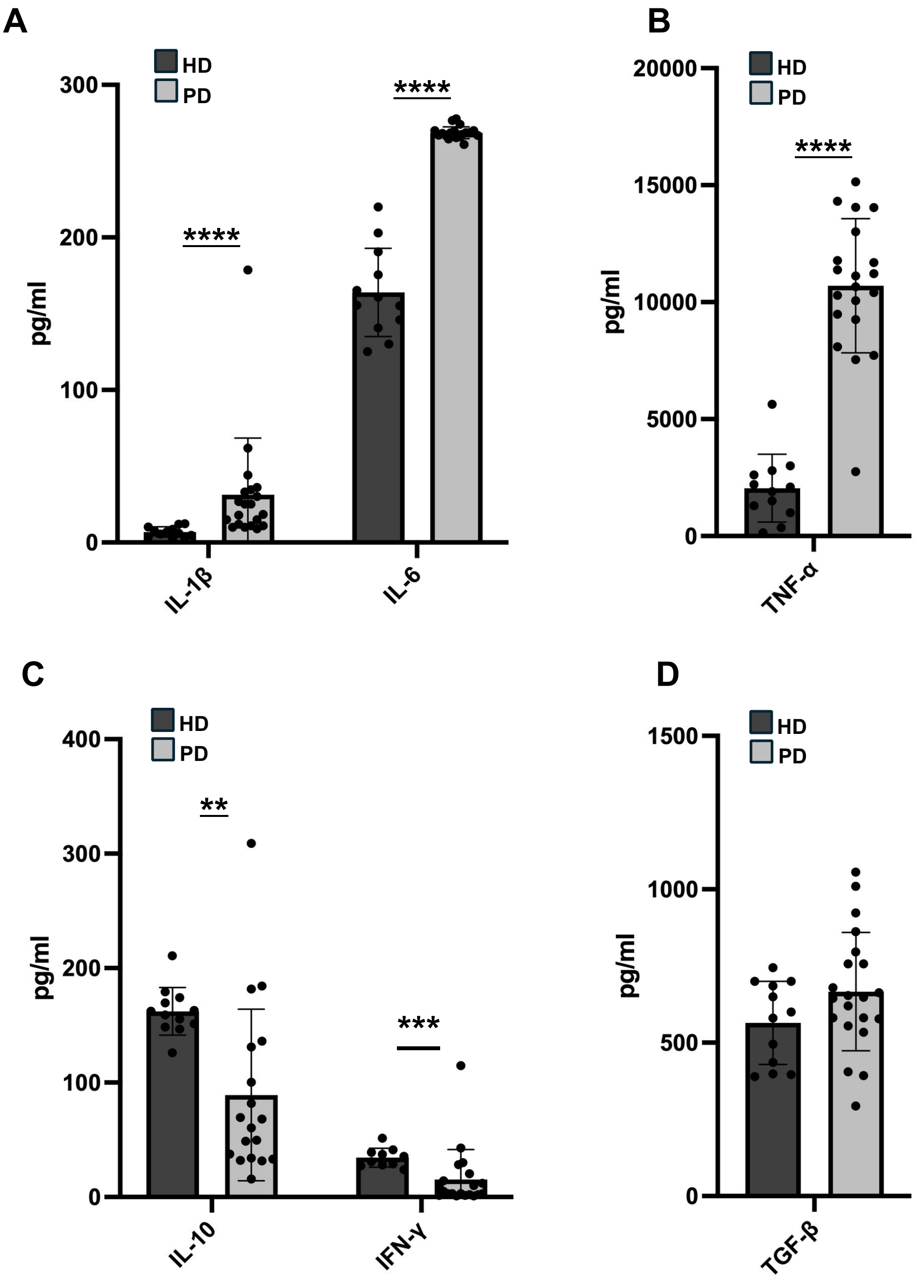

Figure S2 - Supernatants from mature moDCs were analyzed for cytokines levels. Detection of IL-1-β, IL-6 (A), TNF-α (B), IL-10, IFN-γ (C) and TGF-β (D) levels in supernatants of mature DCs derived from monocytes of PD patients (n = 24) and HD (n = 12). Cytokines were quantified by ELISA. Results are expressed as pg/ml.

Data are reported as mean ± SD, ** $p < 0.01$, *** $p < 0.001$, and ****$p < 0.0001$.
