# Supplementary material for: Comparative effect of vortioxetine and sertraline on clinical and inflammatory profile in Parkinson’s disease with comorbid depression
Source: Front Neurosci. 2026 Jan 22;20:1761550. doi: 10.3389/fnins.2026.1761550 (PMC12874709; doi:10.3389/fnins.2026.1761550)
Supplement: Supplementary file 3 [file Data_Sheet_3.pdf]

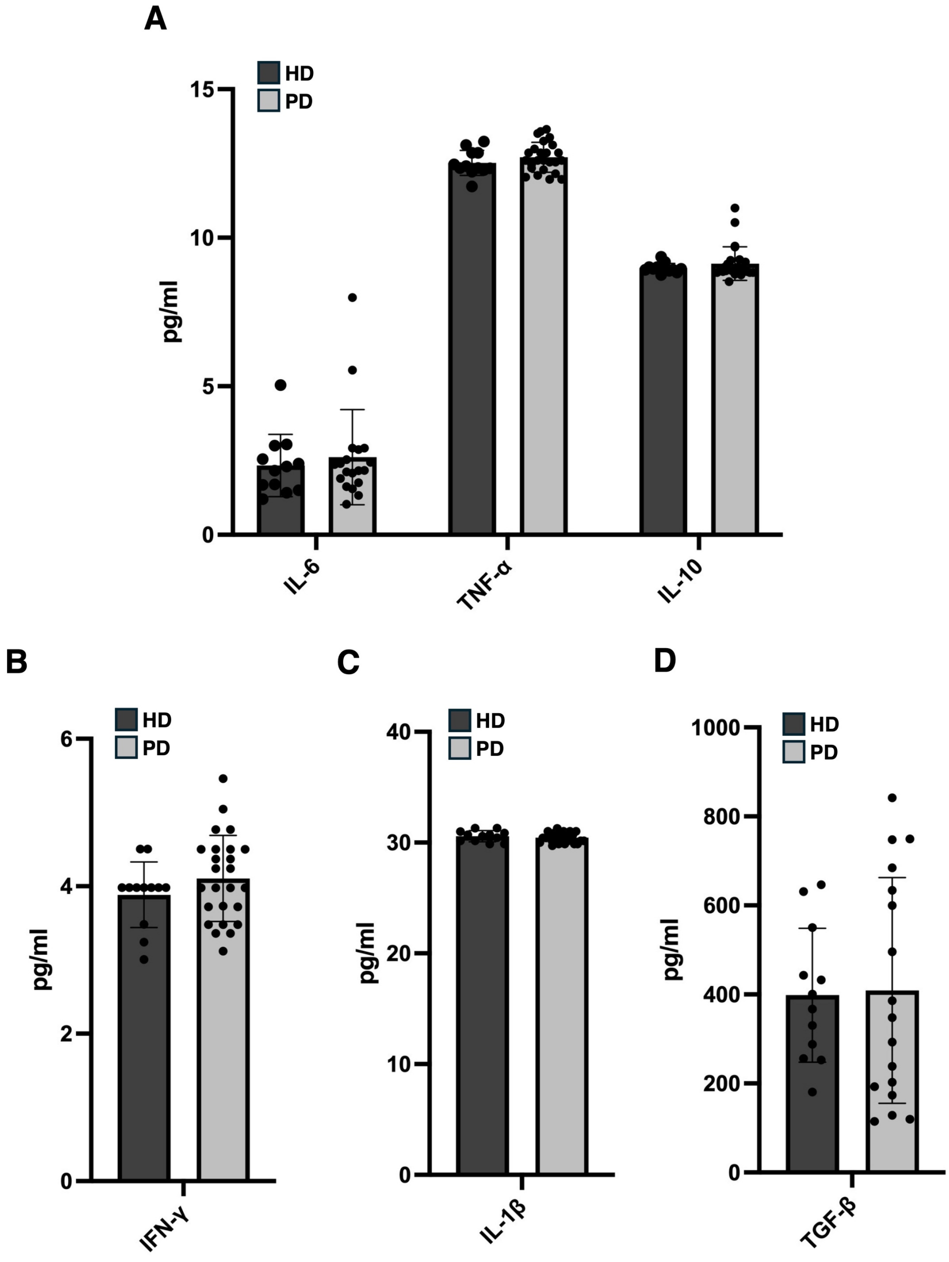

Figure S3 - Plasma of PD patients and HD subjects were analyzed for cytokines levels.

Evaluation of IL-6, TNF-α, IL-10 (A), IFN-γ (B), IL-1-β (C) and TGF-β (D) levels in plasma of PD patients (n = 24) and HD (n = 12), quantified by ELISA. Results are expressed as pg/ml. Data are reported as mean ± SD.
